# Groundwater Pollution Sources Apportionment in the Ghaen Plain, Iran

**DOI:** 10.3390/ijerph15010172

**Published:** 2018-01-22

**Authors:** Mohammad Reza Vesali Naseh, Roohollah Noori, Ronny Berndtsson, Jan Adamowski, Elaheh Sadatipour

**Affiliations:** 1 Department of Civil Engineering, Faculty of Engineering, Arak University, Arak 38156-879, Iran; m-vesalinaseh@araku.ac.ir; 2 Department of Environmental Engineering, Graduate Faculty of Environment, University of Tehran, Tehran 14155-6135, Iran; elahe.sadati@ut.ac.ir; 3 Department of Water Resources Engineering & Center for Middle Eastern Studies, Lund University, Box 118, SE-221 00 Lund, Sweden; ronny.berndtsson@tvrl.lth.se; 4 Department of Bioresource Engineering, Faculty of Agricultural and Environmental Sciences, McGill University, Montréal, QC H3A 0G4, Canada; jan.adamowski@mcgill.ca

**Keywords:** saffron, salinization, health, heavy metals

## Abstract

Although Iran’s Ghaen Plain provides saffron to much of the world, no regional groundwater quality (GQ) assessment has yet been undertaken. Given the region’s potential for saltwater intrusion and heavy metal contamination, it is important to assess the GQ and determine its main probable source of pollution (MPSP). Such knowledge would allow for informed mitigation or elimination of the potential adverse health effects of this groundwater through its use as drinking water, or indirectly as a result of the consumption of groundwater-irrigated crops. Total dissolved solids, sodium, and chloride in the water of the majority of 16 wells sampled within the region exceeded World Health Organization and Iranian permissible standards for drinking water. The groundwater proved to only be suitable for irrigating salt tolerant crops under good drainage conditions. Due to the precipitation of calcium carbonate in the water supply facilities, the water from all wells was deemed unsuitable for industrial purposes. Heavy metal pollution and contamination indices showed no groundwater contamination. Analysis of ionic ratios and the application of principal components analysis indicated the MPSP to be saltwater intrusion, with the geology subtending the plain, and to a lesser extent, anthropogenic activities. Reducing groundwater withdrawals, particularly those for agricultural production by using high performance irrigation methods could reduce saltwater intrusion and improve GQ in the Ghaen Plain.

## 1. Introduction

In arid and semiarid parts of the world, groundwater is often the only source of water available for agricultural, industrial, and drinking purposes. The deterioration of groundwater quality (GQ) in the saffron-producing Ghaen Plain (South Khorasan Province, Iran) is largely attributable to pollution arising from saltwater intrusion, use of chemical fertilizers, and the disposal of municipal sewage. The Ghaen Plain is situated northeast of the central Dasht-e Lut desert, where the moderate resolution imaging spectroradiometer (MODIS) on NASA’s Aqua satellite detected a maximum land surface temperature of 70.7 °C, making the desert the hottest place on Earth. Shallow saltwater table aquifers beneath the Ghaen Plain have rendered the groundwater unsuitable for drinking and irrigation; notwithstanding, its continued use has led to decreasing groundwater levels, raising the risk of saltwater intrusion from the Dasht-e Lut desert. Uncontrolled use of chemical fertilizers for agricultural purposes and infiltration of municipal wastewaters into groundwater due to a lack of sewage systems may have further reduced its GQ and made it potentially hazardous to drink or use in the irrigation of agricultural crops. The Ghaen Plain may also be affected by heavy metals, which could result in significant ecological problems and bio-accumulation through the food chain. These elements are highly toxic, even at low levels, and as they are non-degradable, they have a lengthy residence time in the environment [1,2,3,4]. Accordingly, determining the main probable source of pollution (MPSP) in the Ghaen Plain is important to anticipate the potential health effects on the region’s inhabitants arising from drinking and irrigating with this groundwater. 

Ionic ratios and statistical analyses, particularly principal components factor analysis (PCFA), have been used in several studies to determine the MPSP of water resource systems or individual water bodies [5,6,7,8,9,10,11,12,13]. While some studies have determined the MPSP of groundwater using only ionic ratios [14,15,16,17,18,19,20,21], given the large margins of error for both ionic ratio and PCFA methods’, it is more accurate to apply both methods simultaneously to determine MPSP. If both methods concur, findings are deemed more reliable for planning and management strategies targeted at protecting the GQ. Accordingly, some studies have applied both methods in determining the MPSP of groundwater [22,23,24]. 

Although the Ghaen Plain plays a vital role in providing saffron to the world, neither an assessment of its GQ, nor an evaluation of the potential hazard of saltwater intrusion and heavy metals have been made for the region. Therefore, the main objectives of this study were to: (*i*) Conduct a field study of the physiochemical parameters of groundwater (PCPs) in the Ghaen Plain; (*ii*) Compare observations with permissible levels; (*iii*) Evaluate heavy metal pollution in groundwater by means of the heavy metal pollution index (HPI) and the contamination index (C_d_); and, (*iv*) Identify groundwater MPSP through the application of ion ratios and PCFA.

## 2. Materials and Methods 

### 2.1. Study Area and Data Collection

**Figure 1:**
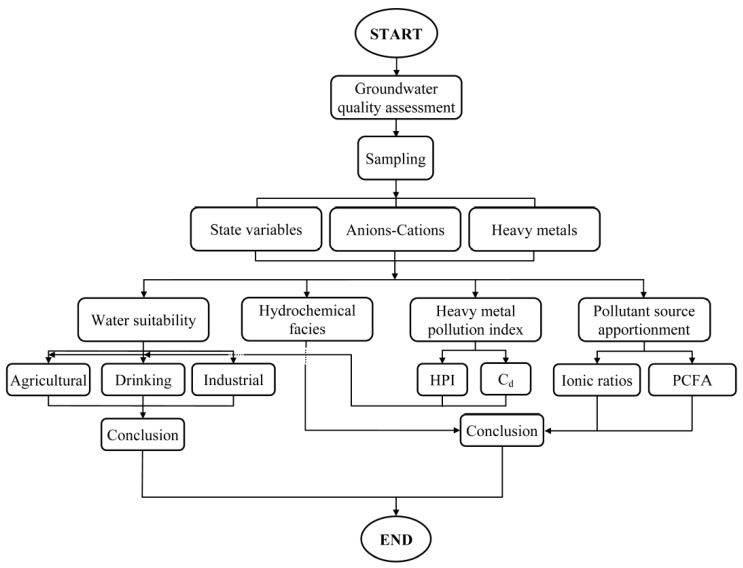
Steps taken in this study to evaluate groundwater quality in the Ghaen Plain.

Steps involved in the evaluation of GQ in the Ghaen Plain are shown in Figure 1.

**Figure 2:**
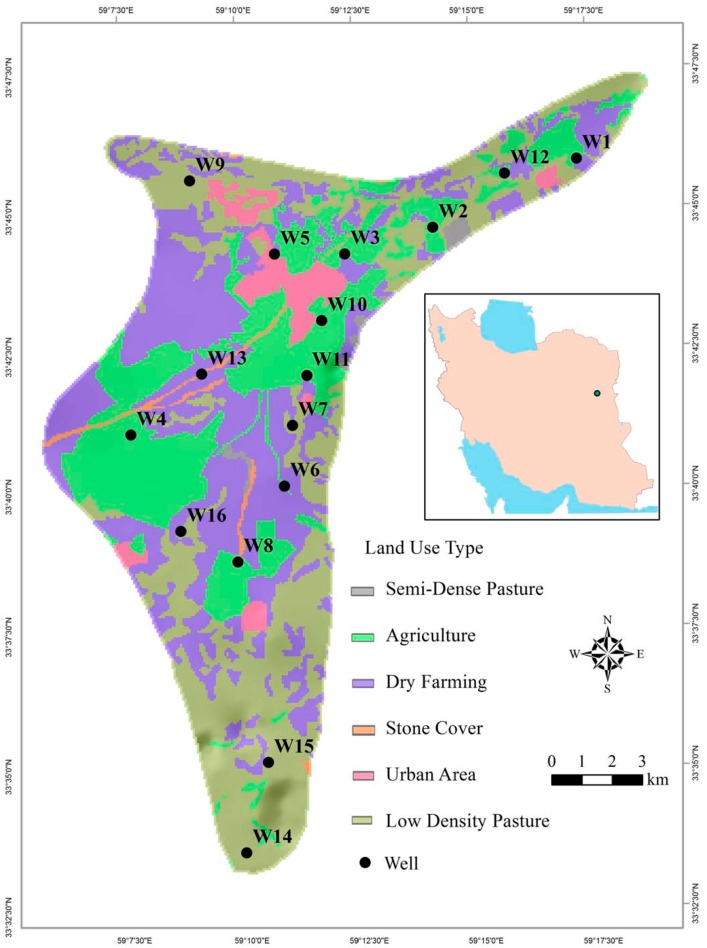
Location of wells sampled on the Ghaen Plain.

The Ghaen Plain is located between 58°34′ and 59°11′ E longitude and between 33°03′ and 33°26′ N latitude (Figure 2). A high rate of evaporation (≈3100 mm year^−1^), low precipitation (≈160 mm year^−1^), lack of perennial rivers and increased demand for domestic consumption and irrigation have caused the groundwater supply to be severely stressed in recent decades.

**Figure 3:**
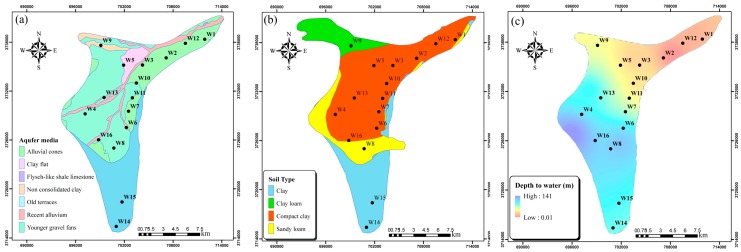
(**a**) Aquifer media; (**b**) soil type; and, (**c**) depth to water in the Ghaen Plain.

The Ghaen aquifer consists mainly of sand mixed with silt and clay, and lesser amounts of glacial deposits, shales, and igneous rocks (Figure 3a). Soils in most parts of the plain are sandy loams, clay and compact clays, and there are some smaller patches of clay loam (Figure 3b). Depth to groundwater in the Ghaen Plain varies from 0 to 140 m and over most of its extent exceeds 30 m (Figure 3c). Since the soil and aquifer consist mainly of fine-grained alluvial deposits and the groundwater in most parts of the plain (except the northeast) is generally deep, it is likely not very exposed to contamination from anthropogenic activities.

Following the guidelines of the *Standard Methods for the Examination of Water and Wastewater* [25], different groundwater samples (metal vs. non-metal analyses) were collected in different numbers at 16 different Ghaen Plain well locations over the 2015 summer season (Figure 2). Water samples were collected in two-liter plastic (non-metals) or glass (metals) vessels. Necessary preservatives were added to samples, and they were placed into a cooler with ice for immediate delivery to the laboratory. Details on holding times and preservations for water samples are given in Table 1. Twenty eight PCPs including: state variables {pH, electrical conductivity (EC), total dissolved solids (TDS), total hardness (TH), total phosphorous (TP), total nitrogen (TN), ammonia (NH_3_), and alkalinity (Alk)}; anions and cations {bicarbonate (HCO_3_^−^), sulfate (SO_4_^2−^), chloride (Cl^−^), orthophosphate (PO_4_^3−^), nitrate (NO_3_^−^), nitrite (NO_2_^−^), fluoride (F^−^), calcium (Ca^2+^), magnesium (Mg^2+^), sodium (Na^+^), potassium (K^+^), and silicon (Si^4+^)}; and heavy metals {boron (B), copper (Cu), chromium (Cr), manganese (Mn), zinc (Zn), iron (Fe), lead (Pb), and cadmium (Cd)} were analyzed for each sample. 

Before sampling, well pumps worked for at least one hour and sampling vessels were rinsed three times with the pumped water, to guarantee representative groundwater samples. The EC, pH, and TDS were analyzed in-situ using a Hach pH meter, whereas other parameters were analyzed in the laboratory. Heavy metals, anions-cations, and TDS-TH were determined by inductively coupled plasma optical emission spectrometry (ICP-OES), ion chromatography (IC), and spectrophotometer DR4000, respectively. A blank sample was tested for each well to determine the accuracy of analyses. Laboratory instrument replicates were used to determine precision for the instruments. Finally, a relative error less than ±5% was achieved for all PCPs analyzed.

### 2.2. Heavy Metal Pollution Indices 

The HPI proposed by Mohan et al. [26] and the C_d_ developed by Backman et al. [27] are useful tools for assessing water quality and can provide practical information for relevant decisions related to the health risks of water pollution. The HPI, calculated according to Equation (1), determines the general status of water quality regarding heavy metals, based on the weighted arithmetic quality mean method:

(1) $$\mathrm{HPI}=\sum_{i=1}^{n}W_{i}Q_{i}/\sum_{i=1}^{n}W_{i}$$

where, *W_i_* (unit weightage) is calculated as inversely proportional to the standard permissible value of the corresponding parameter [28], *n* is the number of parameters, and *Q_i_* is the sub-index of *i*th parameter, calculated for each parameter:

(2) $$Q_{i}=\sum_{i=1}^{n}\left\{\left[\left|M_{i}-I_{i}\right|/\left(S_{i}-I_{i}\right)\right]\times 100\right\}$$

where *M_i_* is the measured *i*th parameter, *I_i_* and *S_i_* are the highest desirable and the standard permissible values of the *i*th parameter, respectively. The suggested critical value of HPI is 100 [28]. In the present study, Fe, Cd, Cr, Cu, Mn, Pb, and Zn were considered in calculating the HPI.

The C_d_ denotes the collective contamination effects of several heavy metals [27]:

(3) $$C_{d}=\sum_{i=1}^{n}\left[\left(C_{A_{i}}/C_{N_{i}}\right)-1\right]$$

where $C_{A_{i}}$ and $C_{N_{i}}$ represent the analytical value and upper permissible concentration of the *i*th component, respectively. Note that $C_{N_{i}}$ is equal to *S_i_* in the HPI formula. Based on the C_d_ index, values less than 1, 1 to 3, and more than 3 indicate low, medium, and high levels of heavy metal contamination, respectively. 

### 2.3. Principal Components Factor Analysis (PCFA)

PCFA can be used to identify MPSP in water resource systems [29,30]. To determine the MPSP in the Ghaen aquifer, the 28 PCPs measured in the wells were used as primary variables for application of the PCFA (*X*_1_, *X*_2_, …, *X*_28_). Thereafter, these primary variables were transferred to 28 independent principal components (PCs) (*ξ*_1_, *ξ*_2_, …, *ξ*_28_). By using PCFA, each PC was expressed as a linear combination of the 28 PCPs [31]:

(4) $$\xi_{i}=\psi_{i1}X_{1}+\psi_{i2}X_{2}+\cdots +\psi_{i28}X_{28}$$

where, $\psi_{i1}$ are eigenvectors extracted from the eigenvalue problem [32]:

(5) |Γ−δI|=0

where, *I* = unit matrix, Г = correlation matrix among 28 PCPs, and δ = eigenvalues [33]. Eigenvectors corresponding to the eigenvalues can be calculated by applying Equation (5). Since this transformation is orthogonal from *X* to *ξ*, Equation (4) can be rewritten as [34]:

(6) $$X_{i}=\psi_{1i}\xi_{1}+\psi_{2i}\xi_{2}+\cdots +\psi_{28i}\xi_{28}$$

Since the few first PCs represent most of the variance of the input variables, only the first *k* PCs are used [35]. Given this assumption, Equation (6) can be expressed as:

(7) $$X_{i}=\sqrt{\delta_{1}}\psi_{1i}\Theta_{1}+\sqrt{\delta_{2}}\psi_{2i}\Theta_{2}+\cdots +\sqrt{\delta_{k}}\psi_{ki}\Theta_{k}+l_{i}$$

where, *l* is a linear combination of Θ*_k+_*_1_ to Θ_28_ PCs, and Θ*_k_* is the principal factors calculated as $\Theta_{k}=\xi_{k}/\sqrt{\delta_{k}}$.

The PCFA model without rotation is expressed as [34]:

(8) $$X_{i}=\beta_{i1}\Theta_{1}+\beta_{i2}\Theta_{2}+\cdots +\beta_{ik}\Theta_{k}+l_{i}$$

where *β_ij_* can be calculated as $\beta_{i}{}_{j}=\sqrt{\delta_{j}}\psi_{ji}$.

The final step in the application of PCFA is the implementation of a VARIMAX rotation on the eigenvectors matrix [36,37]:

(9) $$\Theta^{*}=XG{(G^{\prime }G)}^{-1}$$

where $\Theta^{*}$ is the rotated factor matrix, **X** is the main data matrix which contains mean and variance equal to zero and one, respectively, and **G** is the matrix of factor loadings ($\vartheta_{ik}$). Finally, Equation (9) can be rewritten as:

(10) $$\Theta_{i}^{*}=\vartheta_{1i}X_{1}+\vartheta_{2i}X_{2}+\cdots +\vartheta_{ki}X_{k}+l_{i}$$

One can now extract the rotated principal factors (RPFs) from the primary data for determination of MPSP in each water body system [34].

## 3. Results and Discussion

### 3.1. Sampling Results

**Figure 4:**
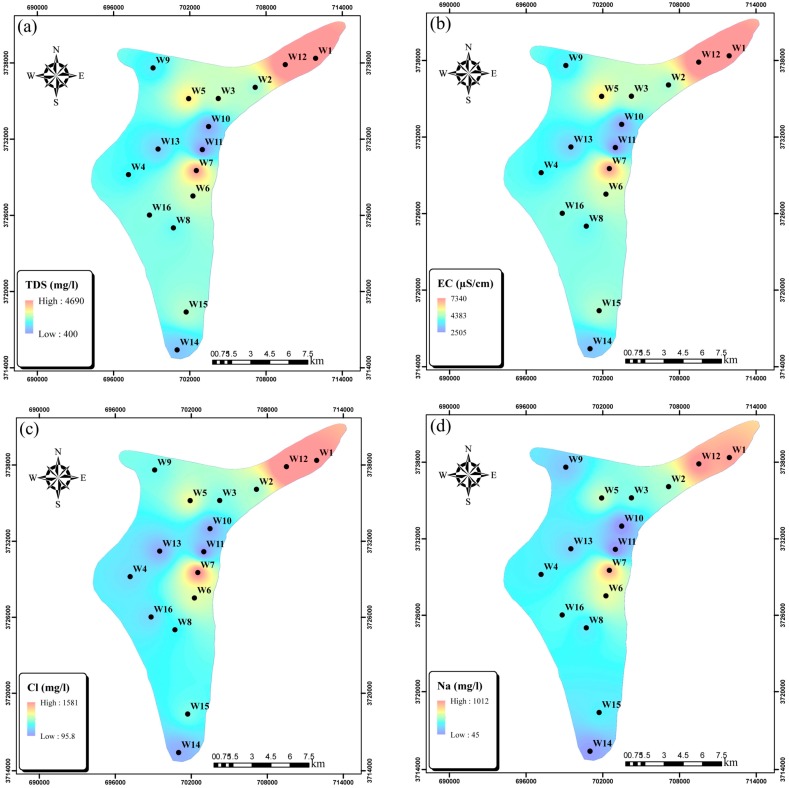
Spatial distribution of (**a**) total dissolved solids (TDS); (**b**) EC; (**c**) chloride (Cl^−^); and, (**d**) sodium (Na^+^) in the aquifer.

The PCPs measured at sampling wells along with their main statistical indices are presented in Table S1 of the electronic supplementary material (ESM) and Table 1, respectively. The goodness-of-fit of the collected data to a log-normal distribution was tested using Kolmogorov-Smirnov statistics, which determined that all the measured data were log-normally distributed with a 95% confidence level. The measured PCPs (Table S1) indicate that the main concerns regarding water quality in the Ghaen aquifer are high levels of EC, TDS, Na^+^, and Cl^−^. Their large spatial variation in the aquifer is shown in Figure 4a–d, respectively. Maximum contaminant concentrations can be seen in the northeastern part of the plain.

### 3.2. Groundwater Quality Assessment

#### 3.2.1. Drinking Water Suitability

**Figure 5:**
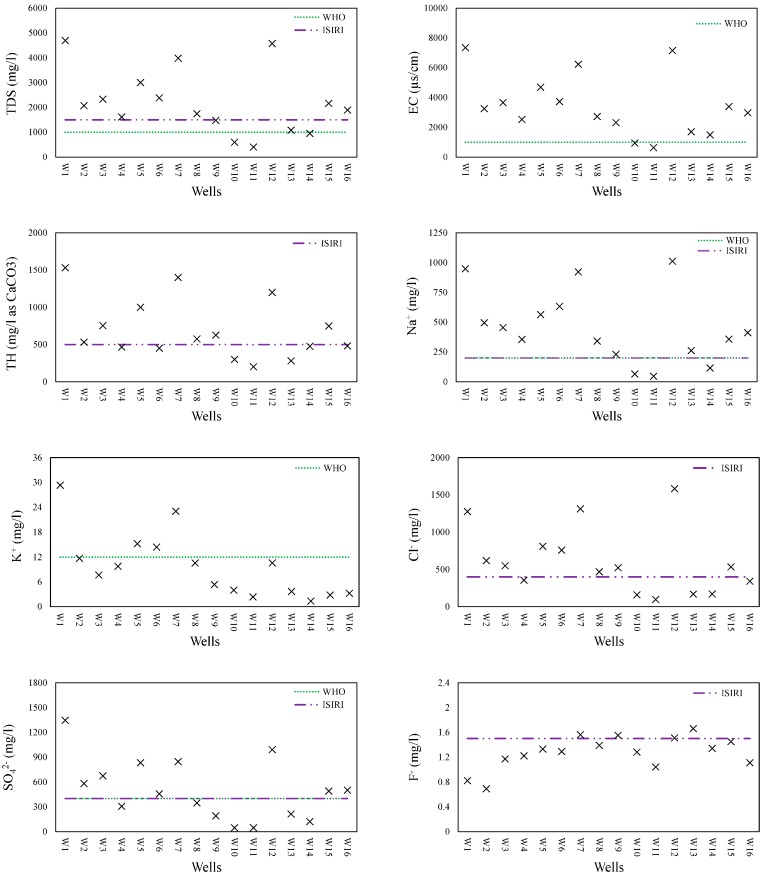
Groundwater quality parameters beyond the permissible limits suggested by the Iranian standard for drinking water (ISIRI) and World Health Organization (WHO).

All PCPs were compared with Iranian standards for drinking water (ISIRI) and those of the World Health Organization (WHO) to ascertain the suitability of GQ in the study area for drinking purposes. While most of the PCPs measured (Table S1) were within permissible limits for drinking water, those that were not are illustrated in Figure 5. 

Although all wells had high concentrations of Mg^+2^, there is no ISIRI- or WHO-recommended limit for Mg^2+^ (Table S1). However, some studies indicate that long term exposure to Mg^2+^ may result in cardiovascular and oncological diseases [38,39,40]. Measured TDS values varied from 400 to 4690 mg L^−1^ with an average of 2183 mg L^−1^, thereby exceeding, in most cases, the ISIRI and WHO maximum permissible levels of 1500 and 1000 mg L^−1^, respectively (Figure 5). The TH in the groundwater samples varied between 201 and 1532 mg L^−1^ as calcium carbonate. Based on the Todd [41] classification, all groundwater samples could be classified as very hard water. Only seven wells had a TH lower than the permissible limit determined by ISIRI (Figure 5). The Cl^−^ concentrations were between 95.9 and 1581 mg L^−1^, with most groundwater samples exceeding ISIRI’s 400 mg L^−1^ permissible limit (Figure 5). There were few health-related concerns regarding K^+^ and F^−^ in the groundwater because concentrations in most wells were below permissible WHO and SIRI limits. However, Well 1 had a K^+^ concentration about 2.5 times higher than permissible levels (Figure 5). 

The high concentrations of some PCPs, such as Mg^2+^, Cl^−^, and TH, and their potential human health effects [42,43], indicate that additional research should be carried out to properly investigate the relationship between exposure to these PCPs and cardiovascular and oncological diseases among the Ghaen Plain’s inhabitants.

#### 3.2.2. Agricultural Water Suitability

**Figure 6:**
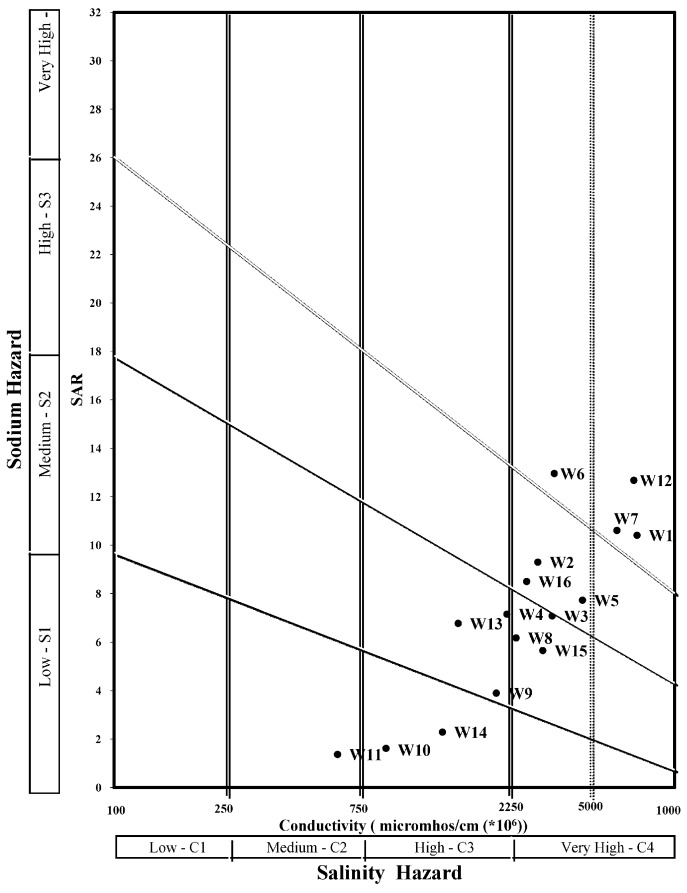
Wilcox diagram for classification of irrigation water in the Ghaen Plain.

Sodium adsorption ratio (SAR), EC, residual sodium carbonate (RSC), and B are the main factors that determine the suitability of water for agricultural purposes. The Wilcox diagram [44], based on SAR and EC, classifies the suitability of water for agricultural purposes in four classes, from excellent (C1–S1) to unsuitable (C4–S4). As illustrated in Figure 6, most groundwater samples fell into the C4–S2 and C4–S3 classes. This indicates very high salinity and medium to very highly sodic water, which would restrict its use for irrigation. Salt tolerant crops under good drainage conditions could be irrigated with this kind of water. Agricultural productivity is directly affected by water salinity and necessary management strategies should be applied if agricultural managers aim to enhance saffron production in the Ghaen Plain.

The RSC, as proposed by Eaton [45], is related to the presence of high concentrations of bicarbonates that precipitate Ca^2+^ and Mg^2+^ from water, cause an increase of Na^+^ in the water in the form of sodium carbonate, and may affect crop yields. Considering that RSC <1.25 meq L^−1^, 1.25 ≤ RSC ≤2.50 meq L^−1^, and RSC > 2.50 meq L^−1^ are considered safe, marginal, or unsuitable for irrigation, respectively, the fact that all well water RSCs values were below 1.25 indicates that the groundwater was safe for irrigation based on this index (Table S1). Since these results contradict the results obtained through the Wilcox diagram, it is important to note that the Wilcox classification is prioritized in making a decision regarding water suitability for irrigation.

Although B is toxic for crops at high concentrations, it is responsible for the transfer of nutrients and water in plants and crop yields are highly affected when soils are B deficient. McCarthy and Ellery [46] suggested limits of B in water for agricultural purposes (Table 2). Based on this classification, B concentrations in all samples were lower than the suggested limit for irrigation of semi-sensitive crops and, accordingly, there is no toxic effect of B in the groundwater of the study area.

#### 3.2.3. Industrial Water Suitability

To determine the suitability of the groundwater for industrial purposes, the saturation index proposed by Rhades and Bernstein [47] was used. For water samples, the saturation index is calculated as the difference between measured pH (pH_w_) and calculated pH (pH_cal_):

(11) $$\mathrm{Saturation}\;\mathrm{Index}=\left({\mathrm{pH}}_{w}-{\mathrm{pH}}_{\mathrm{cal}}\right)$$

(12) $${\mathrm{pH}}_{\mathrm{cal}}=1.776-\log\left({\mathrm{Ca}}^{2+}\right)-\log\left(\mathrm{Alk}\right)$$

where, Ca^2+^ is the molar concentration of Ca^2+^, and Alk is the equivalent concentration of carbonate and bicarbonate. Positive values of the saturation index (Table S1) indicating precipitation of calcium carbonate, tagged water from all wells as being unsuitable for industrial applications. While no major industries currently draw upon Ghaen Plain groundwater, should this occur, the water would have to be pretreated before use.

### 3.3. Hydrochemical Facies

**Figure 7:**
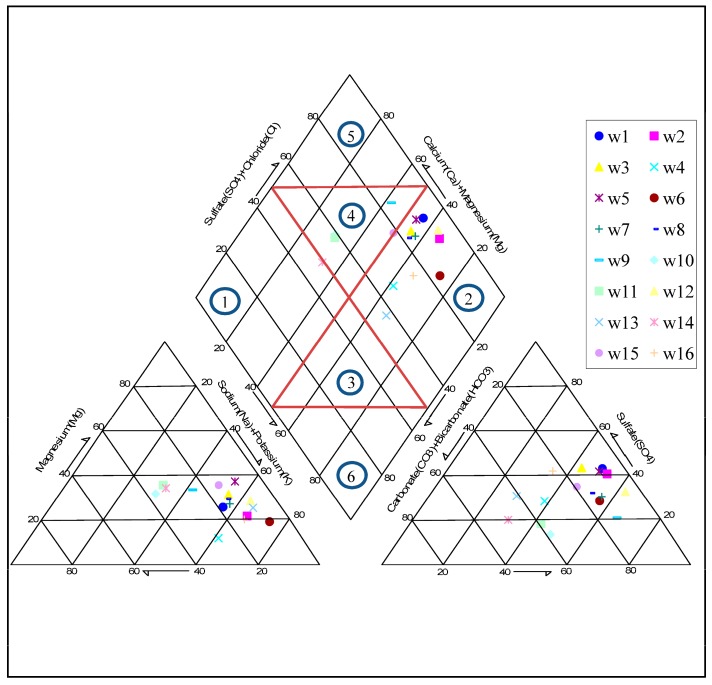
Piper diagram for identification of water type and hydrochemical facies of the Ghaen Plain groundwater.

Consisting of two triangular and one diamond-shaped field, the Piper diagram [48] is widely used to represent the hydrochemical facies of groundwater. Accordingly, a Piper diagram was plotted to identify the water type and hydrochemical regime of groundwater in the Ghaen Plain (Figure 7). For most samples, Na^+^ and Cl^−^ were the predominant cation and anion, respectively. The diamond-shaped part of the diagram is divided into six classes: (1) CaHCO_3_ type; (2) NaCl type; (3) mixed CaNaHCO_3_; (4) mixed CaMgCl; (5) CaCl type; and, (6) NaHCO_3_ type. The most dominant class in the groundwater of the Ghaen Plain was the NaCl type, while the next most important was the mixed CaMgCl type. These findings may reflect saltwater intrusion.

### 3.4. HPI and C_d_ Results

**Figure 8:**
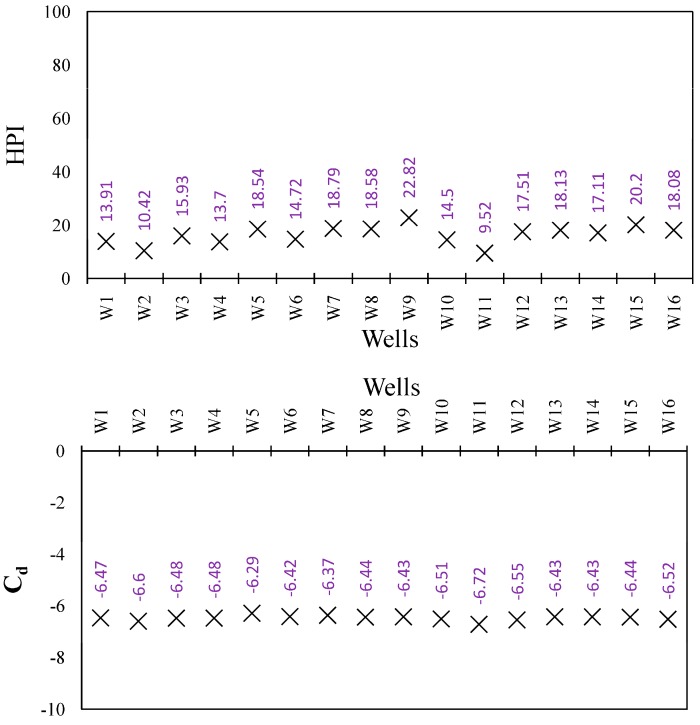
Heavy metal pollution index (HPI) and contamination index (C_d_) for each well in the plain.

Compared in Table S1, measured levels and ISIRI/WHO permissible limits of Fe, Zn, Mn, B, Cr, Cu, Pb, and Cd, show all heavy metals to be below permissible limits, indicating that there is no concern regarding heavy metal contamination of the groundwater. All HPI values were below the critical value (100) and all C_d_ values were below 1 (Figure 8), indicating a low level of heavy metal contamination. Therefore, there is no concern regarding heavy metal pollution for drinking water or agricultural purposes.

### 3.5. The MPSP

#### 3.5.1. Identifying the MPSP by Ionic Ratios

• Mg^+2^/Ca^+2^ Ratio

**Figure 9:**
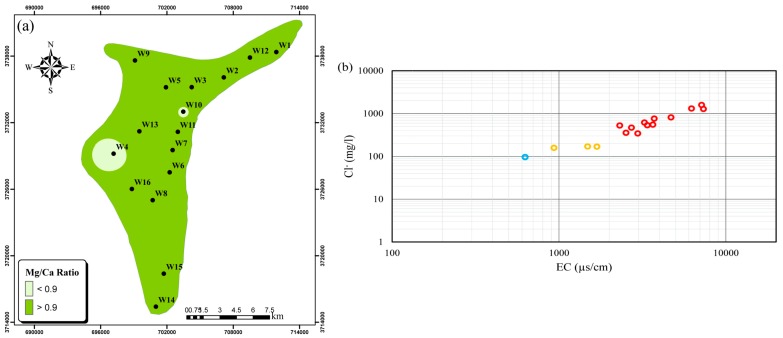
Spatial distribution of (**a**) magnesium (Mg^+2^)/calcium (Ca^+2^) ratio and (**b**) Cl^−^ vs. EC diagram in the plain.

The cations Ca^+2^ and Mg^+2^, belonging to the most abundant of the alkaline-earth metals, are major constituents of most freshwater systems. Although Ca^+2^ concentrations in all groundwater samples were below the permissible 300 mg L^−1^, all wells had high Mg^2+^ concentrations (Table S1). As it has been suggested that a Mg^+2^/Ca^+2^ ratio exceeding 0.9 indicates saltwater intrusion [49,50], and that this ratio value was exceeded in 14 of 16 wells, saltwater intrusion may pose an important threat to groundwater in the Ghaen Plain. The spatial distribution of the Mg^+2^/Ca^+2^ ratio in the Ghaen aquifer (Figure 9a) reveals that most parts of the study area, especially the northeast, may be exposed to saltwater intrusion. 

• Cl^−^/EC Ratio

The Cl^−^/EC ratio has been suggested as a good indicator of saltwater intrusion by the Washington State Department of Ecology [51]. A graphical approach plotting Cl^−^ vs. EC (Figure 9b) shows three zones: normal, mixed, and saltwater intrusion. The Cl^−^ and EC in most groundwater samples exceeded 200 mg L^−1^ and 1000 µs cm^−1^, respectively (red circles) and are most likely influenced by saltwater intrusion [51]. Samples W10, W13, and W14 are characterized by Cl^−^ levels between 100 and 200 mg L^−1^ and EC between 600 and 2000 µs cm^−1^ (yellow circles), which represent a mixing of freshwater and saltwater [51]. Sample W11, characterized by Cl^−^ and EC lower than 100 mg L^−1^ and 600 µs cm^−1^, respectively, represents normal freshwater (blue circle) [51]. Therefore, it can be concluded that saltwater intrusion may pose a major threat to groundwater in the Ghaen Plain.

• Simpson’s Ratio (Cl^−^/HCO_3_^−^ + CO_3_^−2^)

**Figure 10:**
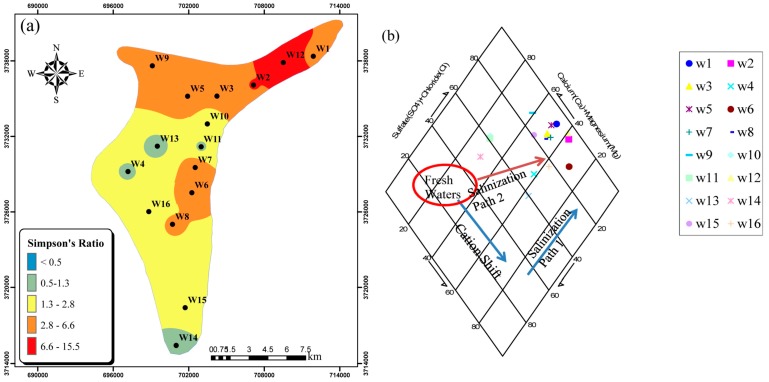
Saltwater intrusion based on (**a**) Simpson’s ratios and (**b**) Piper diagram for the aquifer.

Simpson’s ratio can also be used to determine the extent of saltwater intrusion in groundwater [16,41]. On the basis of this ratio, groundwater is classified into five classes: good quality (<0.5), slightly contaminated (0.5 to 1.3), moderately contaminated (1.3 to 2.8), contaminated to a major extent (2.8 to 6.6), and highly contaminated (6.6 to 15.5) [41,52]. The distribution of the Simpson’s ratio over the study area shows the northeastern portions of the study area to be contaminated to a major extent or highly contaminated (Figure 10a). This again indicates the influence of saltwater intrusion into the Ghaen aquifer.

• Piper Diagram

The diamond shape of the Piper diagram can serve to determine the possibility of saltwater intrusion in groundwater. Allen and Suchy [14] suggested that two paths shown in the diamond shape of the Piper diagram (Figure 10b) indicate salinization of groundwater by saltwater intrusion. Figure 10b clearly shows that most groundwater samples in the study are exposed to saltwater intrusion.

#### 3.5.2. Identifying the MPSP by PCFA

PCFA was applied to determine MPSP for the Ghaen aquifer. The correlation matrix Г was calculated with eigenvalues, along with eigenvectors corresponding to the calculated RPFs obtained by considering the VARIMAX rotation. Table 3 shows the corresponding eigenvalues and percentage of variance explained by each RPF obtained by the application of PCFA. Given the few first eigenvalues greater importance, PCFA results were considered for only the first 6 of 28 RPFs exceeding one. About 86% of the variance in data were explained by the first six RPFs (Table 4). Factor loadings that exceeded 0.90 were considered significant for the selected RPFs. Only the first three RPFs had factor loadings greater than 0.9 (Table 4).

The EC, TDS, TH, Mg^2+^, Na^+^, SO_4_^2−^, and Cl^−^ were the PCPs with greatest influence on the first factor (Table 4). The EC is related to the ionic content of the samples, which in turn, is a function of TDS concentration. The TDS, EC, and major ions in Ghaen Plain groundwater most likely originated from saltwater intrusion and the study area’s geologic structure [53]. Moreover, Cl^−^ can originate from either natural or anthropogenic sources [54]. First factor results indicate that both Cl^−^ and other major ions should have the same origin (natural source). Chloride is among the main PCPs related to salinization; its levels exceeding 100 mg L^−1^ indicate saltwater intrusion [55]. This ion in conjunction with Na^+^ composes sodium chloride. The issue of high saltwater content has been reported by regional drinking water authorities in recent years. The presence of Mg^2+^ in conjunction with SO_4_^2−^ makes up magnesium sulphate, which has medical implications when found in drinking water. By considering these findings and the ionic ratios, all PCPs can be concluded to indicate saltwater intrusion into the Ghaen aquifer, aided by the region’s geologic structure. 

The second factor was mostly strongly influenced by PO_4_^3−^, NO_3_^−^, and TP levels (Table 4). As these parameters are characteristic of eutrophication in surface waters, they most probably relate to contamination from agricultural activities and pit latrines used for disposal of wastewater in the study area. Generally, overuse of manure and fertilizers on agricultural lands, absence of wastewater collection systems, and the use of pit latrines introduces a high volume of nutrient loading into the aquifer. 

The third factor is highly influenced by Alk and HCO_3_^−^ (Table 4), which originate from the CO_2_ gas fraction of the atmosphere, or the atmospheric gases present in the soil in the unsaturated zone [49]. In the study area, Alk and HCO_3_^−^ likely originate from silicate weathering from the surface soils due to the high alkalinity in the plain and groundwater salinization from saltwater intrusion. Therefore, similar to the ionic ratio results, the PCFA analysis suggests that saltwater intrusion, and the plain’s geologic structure, can be considered as the MPSP for the aquifer.

## 4. Conclusions

In the present study, the GQ of the Ghaen Plain was investigated by analyzing the PCPs and heavy metal concentrations in 16 wells. High levels of TDS, Cl^−^, Na^+^, and Mg^2+^ in the groundwater revealed some problematic issues for its use as drinking or irrigation water. Saltwater intrusion and the geology underlying the study area are the main sources of these problems. All heavy metal levels in the groundwater were within the ISIRI and WHO permissible levels for drinking water and these results are supported by the measured HPI and C_d_ values. High levels of EC, which resulted in classification of most well water samples as very high saline and medium to very high in sodium indicate that the groundwater is suitable only for irrigation of salt tolerant crops under good drainage conditions. Due to the positive saturation index, which indicates precipitation of calcium carbonate in water supply facilities, the water from all wells was deemed unsuitable for industrial purposes. The Mg^+2^/Ca^+2^ ratio, Simpson ratio, Cl^−^ vs. EC diagram, Piper diagram, and PCFA showed that saltwater intrusion can be considered an MPSP in the Ghaen aquifer. The PCFA results showed that there were three MPSPs in the study area: saltwater intrusion, geology underlying the Ghaen aquifer, and anthropogenic sources, such as agriculture and wastewater disposal through septic wells. 

Since more than 90% of Iran’s water consumption supports agricultural activities, and the Ghaen aquifer’s main MSPS is saltwater intrusion, the use of high performance irrigation methods may be the best alternative to reducing groundwater withdrawals for agricultural purposes and improving groundwater quality in the Ghaen Plain. Based on the geological properties and the area of the plain, a higher number of sampling points might better delineate these MPSPs. It should be noted that the authors are familiar with the study area’s characteristics, such as geological structure, land use, pollution sources, and constraints for accessing some of the wells. In this regard, the sampling points were selected in a representative manner as regards changes in the water quality status in the aquifer.

## Figures and Tables

**Table 1:** Summary statistics for groundwater in the Ghaen Plain.

Parameters	Unit	Mean	Median	SD *	Minimum	Maximum	Preservation	Holding Time
pH	Standard	7.49	7.5	0.32	7.09	8.11	-	ISM ^†^
EC	µs cm^−1^	3416	3108	2040	627	7340	-	ISM ^†^
TDS	mg L^−1^	2183	1981	1304	400	4690	-	ISM ^†^
TH (as CaCO_3_)	mg L^−1^	688	553	398	201	1532	Cool, 4 °C ^‡^	24 h
Alk (as CaCO_3_)	mg L^−1^	274	281	104	124	480	Cool, 4 °C ^‡^	24 h
Ca^2+^	mg L^−1^	112.4	96.3	66.6	35.5	292.0	Cool, 4 °C ^‡^	24 h
Mg^2+^	mg L^−1^	99.5	79.1	66.1	24.0	210.0	Cool, 4 °C ^‡^	24 h
Na^+^	mg L^−1^	450.6	384.4	302.7	45.0	1012.0	Cool, 4 °C ^‡^	24 h
K^+^	mg L^−1^	9.7	8.7	7.9	1.3	29.3	Cool, 4 °C ^‡^	24 h
Si^4+^	mg L^−1^	2.3	2.5	0.8	1.0	3.9	Cool, 4 °C ^‡^	24 h
HCO_3_^−^	mg L^−1^	335.0	336.8	126.7	152.5	585.1	Cool, 4 °C ^‡^	24 h
SO_4_^2−^	mg L^−1^	498.5	471.8	367.1	45.3	1345.0	Cool, 4 °C ^‡^	24 h
Cl^−^	mg L^−1^	607.5	528.6	445.1	95.9	1581.0	Cool, 4 °C ^‡^	24 h
PO_4_^3−^	mg L^−1^	0.13	0.1	0.04	0.07	0.22	Cool, 4 °C ^‡^	24 h
NO_3_^−^	mg L^−1^	16.88	15.7	5.61	7.11	28.9	Cool, 4 °C ^‡^ and pH below 2 by H_2_SO_4_	72 h
NO_2_^−^	mg L^−1^	0.02	0.01	0.01	0.009	0.039	Cool, 4 °C ^‡^	24 h
NH_3_	mg L^−1^	0.03	0.01	0.01	0.013	0.055	Cool, 4 °C ^‡^	24 h
F^−^	mg L^−1^	1.28	1.3	0.27	0.69	1.66	None required	96 h
B	mg L^−1^	2.07	1.7	1.05	0.56	4.34	Cool, 4 °C ^‡^	24 h
TP	mg L^−1^	0.17	0.2	0.05	0.087	0.26	Cool, 4 °C ^‡^ and pH below 2 by H_2_SO_4_	72 h
TN	mg L^−1^	1.01	0.9	0.35	0.55	1.66	Cool, 4 °C ^‡^ and pH below 2 by HNO_3_	72 h
Fe	µg L^−1^	102.5	89.0	40.2	41.8	187.6	Cool, 4 °C ^‡^ and pH below 2 by HNO_3_	96 h
Zn	µg L^−1^	27.89	24.1	10.43	15.9	55.1	Cool, 4 °C ^‡^ and pH below 2 by HNO_3_	96 h
Mn	µg L^−1^	19.54	20.1	6.90	9.12	33.9	Cool, 4 °C ^‡^ and pH below 2 by HNO_3_	96 h
Cr	µg L^−1^	2.24	2.2	0.68	1.23	3.67	Cool, 4 °C ^‡^	24 h ^‡^
Cu	µg L^−1^	23.97	21.3	11.19	11.5	49.3	Cool, 4 °C ^‡^ and pH below 2 by HNO_3_	96 h
Pb	µg L^−1^	5.54	5.5	2.57	2.09	10.4	Cool, 4 °C ^‡^ and pH below 2 by HNO_3_	96 h
Cd	µg L^−1^	1.09	1.1	0.24	0.67	1.55	Cool, 4 °C ^‡^ and pH below 2 by HNO_3_	96 h

* Standard Deviation (SD); ^†^ In situ measurement (ISM); ^‡^ In a cooler with temperature of ~4 °C.

**Table 2:** Permissible limits of Boron in irrigation water for crops [46].

Boron Class	Range (mg L^−1^)		
	Semi-Sensitive Crops	Semi-Tolerant Crops	Tolerant Crops
Excellent	<0.33	<0.67	<1
Good	0.33–0.67	0.67–1.33	1–2
Permissible	0.67–1	1.33–2	2–3
Doubtful	1–1.25	2–2.5	3–3.75
Unsuitable	>1.25	>2.5	>3.75

**Table 3:** Characteristics of the calculated rotated principal factors.

RPFs	Eigenvalue	PVE * by Each RPF	PCVE ^†^ by Each RPF
RPF 1	9.36	33.4	33.4
RPF 2	5.12	18.4	51.8
RPF 3	3.90	13.9	65.7
RPF 4	2.17	7.7	73.4
RPF 5	1.90	6.8	80.2
RPF 6	1.62	5.8	86.0

* % of Variance Explained (PVE); ^†^ % of Cumulative Variance Explained (PCVE).

**Table 4:** Principal components factor analysis (PCFA) loadings matrix.

Parameter	RPF 1	RPF 2	RPF 3
pH			
EC	0.98		
TDS	0.95		
TH (as CaCO_3_)	0.95		
Alk (as CaCO_3_)			0.94
Ca^2+^			
Mg^2+^	0.92		
Na^+^	0.95		
K^+^			
Si^4+^			
HCO_3_^−^			0.94
SO_4_^2−^	0.96		
Cl^−^	0.96		
PO_4_^3−^		0.91	
NO_3_^−^		0.90	
NO_2_^−^			
NH_3_			
F^−^			
B			
TP		0.91	
TN			
Fe			
Zn			
Mn			
Cr			
Cu			
Pb			
Cd			

## Supplementary Materials

The following are available online at http://www.mdpi.com/1660-4601/15/1/172/s1, Table S1: Physiochemical parameters at sampling sites {in mg L^−1^, except for pH (standard unit), EC (µs cm^−1^), and heavy metals (µg L^−1^)}.
