# Supplementary material for: Groundwater Pollution Sources Apportionment in the Ghaen Plain, Iran
Source: Int J Environ Res Public Health. 2018 Jan 22;15(1):172. doi: 10.3390/ijerph15010172 (PMC5800271; doi:10.3390/ijerph15010172)
Supplement: Supplementary file 1 [file ijerph-15-00172-s001.pdf]

Table S1 Physiochemical parameters at sampling sites {in mg/l, except for pH (standard unit), EC (µs/cm), and heavy metals (µg/l)}

Parameter	W1	W2	W3	W4	W5	W6	W7	W8	W9	W10	W11	W12	W13	W14	W15	W16	Permissible limit*	
																	ISIRI	WHO
pH	7.23	8.01	7.11	7.43	7.55	7.95	7.11	7.35	7.55	7.52	7.57	8.11	7.64	7.09	7.35	7.31	6.5-9	**
EC	7340	3250	3655	2520	4689	3720	6225	2719	2310	934	627	7150	1689	1485	3385	2965	**	1500
TDS	4690	2072	2330	1610	3000	2380	3980	1740	1475	595	400	4570	1080	950	2160	1890	1500	1000
TH (as $CaCO_3$)	1531.6	530	753.3	465	1000	450	1400	575	625.5	300	201	1200	280	475.6	748	480.2	500	**
Alk (as $CaCO_3$)	295.6	124	265.5	432	227	305	479.5	218	160.7	180	125	212.7	335.2	370	325.8	335.2	**	**
Ca^{2+}	292	92	109	142	92.5	60	235.5	90	121	70	42	131.5	35.5	100	100.5	85	**	300
Mg^{2+}	201	73.2	121.2	26.4	185.5	72	201.3	84.9	85.5	31.2	24	210	45.9	55	120.1	55.5	**	**
Na^{+}	948.3	494.5	455	355	563.5	632.5	921.8	341.9	230.5	65	45	1012	261	115.1	356.8	411.9	200	200
K^{+}	29.3	11.7	7.65	9.75	15.2	14.4	23	10.53	5.34	3.98	2.35	10.56	3.67	1.33	2.79	3.21	**	12
Si^{4+}	2.52	2.09	2.88	1.55	3.23	2.67	1.45	3.88	1.96	2.55	0.98	2.88	2.56	1.69	2.49	1.02	**	**
HCO_3^-	358	158.6	315.5	524.6	277.1	372.1	585.1	266.8	196.9	220	152.5	259.5	409.5	453.6	401.5	409	**	**
SO_4^{2-}	1345	580.6	673.4	305	832.6	455	845.6	346.6	189.5	45.5	45.3	989.8	211.6	120.8	488.5	500.9	400	400
Cl^-	1275.5	617.7	549.3	355	811.3	759.5	1311.6	467.8	523.5	158	95.85	1581	168.3	170.3	533.6	341.8	400	**
PO_4^{3-}	0.09	0.07	0.13	0.17	0.14	0.09	0.15	0.19	0.22	0.17	0.14	0.16	0.12	0.074	0.11	0.13	**	**
NO_3^-	12.1	15.3	11.5	15.5	7.11	21.5	18.3	28.9	24.9	19.5	15.8	11.4	17.33	12.9	15.5	22.6	50	50
NO_2^-	0.017	0.012	0.025	0.013	0.017	0.011	0.015	0.039	0.037	0.019	0.011	0.032	0.009	0.015	0.012	0.011	3	3
NH_3	0.029	0.035	0.028	0.041	0.039	0.023	0.019	0.036	0.051	0.045	0.013	0.019	0.055	0.031	0.023	0.027	**	**
F^-	0.82	0.69	1.17	1.22	1.33	1.29	1.56	1.39	1.55	1.28	1.04	1.51	1.66	1.34	1.45	1.11	1.5	**
B	2.66	1.97	1.41	1.19	3.09	2.54	1.77	3.76	4.34	1.11	0.56	0.93	1.67	1.55	2.84	1.65	500	2400
TP	0.12	0.11	0.15	0.21	0.16	0.13	0.18	0.26	0.25	0.21	0.18	0.18	0.15	0.087	0.15	0.16	**	**
TN	1.62	1.23	1.66	1.17	0.95	0.77	1.41	1.15	0.89	0.94	0.65	1.09	0.59	0.55	0.89	0.61	**	**
Fe	151.5	132.4	91.5	76.9	187.6	155.8	86.5	68.6	106.4	83.3	55.6	41.8	81.5	139.8	103.6	77.6	**	**
Zn	30.2	21.9	20.5	23.7	21.5	15.9	24.4	46.7	28.5	32.6	16.8	55.1	21.7	31.6	31.5	23.7	**	**
Mn	23.9	19.7	11.6	9.12	15.7	20.5	29.7	33.9	21.6	19.5	10.4	13.4	20.9	15.3	26.8	20.6	400	**
Cr	1.88	1.34	1.98	1.32	2.43	1.99	2.31	2.98	3.67	1.77	1.23	2.67	2.44	2.09	3.15	2.55	50	50
Cu	15.7	11.5	15.7	14.1	38.9	12.7	49.3	36.3	28.3	19.4	14.8	23.1	15.9	35.6	28.4	23.8	2000	2000
Pb	4.9	2.33	7.9	9.89	9.33	7.56	6.34	5.22	2.67	5.87	2.09	5.76	7.87	4.43	3.12	2.88	10	10
Cd	0.92	0.77	1.14	1.19	1.22	0.88	1.55	0.88	1.23	1.12	0.67	0.89	1.19	1.44	1.15	1.27	3	3
Mg^{+2}/Ca^{+2} Ratio	1.1	1.3	1.9	0.3	3.3	2.0	1.4	1.6	1.2	0.7	1.0	2.7	2.2	0.9	2.0	1.1		
Simpson's Ratio	6.12	6.69	2.99	1.16	5.03	3.51	3.85	3.01	4.57	1.23	1.08	10.47	0.71	0.65	2.28	1.44		
Saturation Index	6.12	6.97	6.60	6.27	6.73	6.79	6.00	6.76	6.76	6.95	7.33	6.60	6.97	6.48	6.53	6.51		
SAR	10.41	9.30	7.09	7.16	7.73	12.96	10.61	6.18	3.90	1.62	1.37	12.68	6.78	2.29	5.66	8.50		
RSC	-25.5	-8.1	-10.3	-0.7	-15.5	-2.9	-18.9	-7.20	-9.90	-2.5	-1.6	-19.8	1.1	-2.1	-8.5	-2.2		

* For drinking purposes; ** No health concern at levels found in drinking water.
